# Comparison of Telomere Length in Age-Matched Primiparous and Multiparous Brahman Cows

**DOI:** 10.3390/ani13142325

**Published:** 2023-07-16

**Authors:** Sydney E. O’Daniel, Kelli J. Kochan, Charles R. Long, David G. Riley, Ronald D. Randel, Thomas H. Welsh

**Affiliations:** 1 Department of Animal Science, Texas A&M University, College Station, TX 77843, USA; sydney.olson@usda.gov (S.E.O.); david.riley@ag.tamu.edu (D.G.R.); 2 Texas A&M AgriLife Research Center, Overton, TX 75684, USA; charles.long@ag.tamu.edu (C.R.L.); r-randel@tamu.edu (R.D.R.); 3 Texas A&M Institute for Genome Sciences and Society, Texas A&M University, College Station, TX 77843, USA; kkochan@tamu.edu

**Keywords:** stress, telomere, parturition, cattle, Brahman

## Abstract

**Simple Summary:**

The productive longevity of cattle is a difficult trait to quantify, but it is believed to be influenced by genetic factors, which, in turn, may be influenced by environmental stressors. One potential biological marker for cow longevity is the length of the telomere (TL), which is the highly plastic nucleic sequence responsible for protecting the chromosomes’ integrity. Stressors negatively affect the TL in many vertebrate species. Stressors are encountered in all segments of beef production, from breeding and calf production through the finishing phase. We assessed whether the number of parities, duration of labor, and raising a calf were related to the TL in age-matched Brahman females that were pregnant for the first or second time. The parity was negatively associated with peripheral blood leukocyte TL in Brahman cows. In addition to its appeal as a selection criterion for longevity, the TL may be an indicator of welfare due to its susceptibility to stress. The mechanisms subserving the bovine telomere dynamics between birth and culling (or survival) constitute a worthy topic for further exploration, particularly in consideration of stressors that are associated with a changing climate.

**Abstract:**

Physiological and psychological stressors have been associated with the attrition of telomeres, which are the protective caps of chromosomes. This study compares the telomere length (TL) in 4-year-old Brahman cows grouped by the first parity (n = 8) and the second parity (n = 11). The cows were bled via jugular venipuncture, weighed, and had their body condition scores recorded at Day −28 prior to calving and at Day + 7 and Day + 28 post-calving. The duration of labor (Dlabor) and parturition ease were recorded. The peripheral leukocytes were isolated, the leukocyte blood count with differential was recorded, and the genomic DNA was extracted. The relative quantity of telomere products, which is proportional to the average TL, was determined via multiplex quantitative PCR using the ratio (T/S ratio) of bovine telomere and β-globulin DNA. Standards of the bovine telomere (10^12^–10^7^ dilution series) and β-globulin (10^9^–10^4^ dilution series) genes were utilized to produce relative copy numbers. The samples were assayed in triplicate and were included if the triplicate C_q_ difference was less than 0.25 cycles. The parity was the fixed effect, and the random effects included the sire and day repeated with the cow as the subject. Statistical significance was not observed in the leukocyte number or type (*p* > 0.1). A reduction in the TL of approximately 9225 telomeric copies was found between Parity 1 and Parity 2 (*p* = 0.02). A trend was found between the TL and Dlabor (*p* = 0.06). The stress of parturition and raising the first calf of a cow’s life may be responsible for TL attenuation. Parity may be considered a stressor of cow longevity.

## 1. Introduction

Telomeres (5′-TTAGGG-3′) are tandem repeat non-coding DNA sequences oriented toward chromosome termini [1,2]. In many vertebrates, these sequences play a crucial role in maintaining chromosome stability during DNA replication [3]. Telomeres are slowly lost over time due to the “end replication problem”; however, this is mitigated by the ribonucleoprotein enzyme telomerase [4]. Despite lengthening mechanisms, the gradual shortening of telomeres has been associated with increased mortality and the occurrence of multiple age-related diseases in humans [5,6,7]. These results led to the hypothesis that the TL could be a reliable proxy for age or the “mitotic clock”; however, the relationship between aging and the TL has proven to be more complex [8,9,10,11]. This complexity can be largely attributed to the high plasticity of the TL and telomerase behavior when exposed to environmental stressors and the resulting cascade of glucocorticoid signaling, as well as the polygenic nature of telomeres themselves [9,10,11]. There is now strong evidence that the telomere length and dynamics are tightly linked to fitness, longevity, and survival in domesticated [10,11,12,13], captive, and wild [14,15,16,17] vertebrate species. Telomeres are considered to (a) be a molecular marker of biological age; (b) represent the “wear and tear” of organismal experiences; and (c) be an important tool that is used to assess the influence of life history events and environmental conditions in wild and domestic vertebrates [9,18,19].

The shortening of average leukocyte TLs measured in mammalian and non-mammalian vertebrates seems to be related to a lower survival rate and potentially to longevity [9,10]. The association with longevity led to the study of cumulative stress on TL as a biomarker of health and animal well-being [18]. Productive longevity or “functional longevity”, as defined by Seeker et al. [10], has remained an elusive trait to quantify in the dairy cattle industry. The heritability of functional longevity was estimated to be low, approximately 0.01–0.06, until a longitudinal study of 308 Holstein Friesian dairy cows found the heritability estimates of the relative leukocyte telomere length (RLTL) to be 0.38 ± 0.03 and 0.32 ± 0.08 among the cow and calf data sets [10,20]. Within those studies, it was concluded that the RLTL measured between one and five years of age was significantly and positively correlated with the productive lifespan [10]. This was previously suggested in a study that found that dairy cattle with short telomeres were more likely to be culled for poor health or fertility [12]. A further investigation by Ilska-Warner et al. [11] found that the TL measured at birth has a high genetic correlation with survival to 48 months of age and is significantly associated with the length of productive life and the future health status. Using the leukocyte TL as a potential selection criterion for breeding programs was considered due to its linkage to survivability and “functional longevity” in dairy cattle; however, the TL may vary between breeds [21,22,23].

A biomarker for productive longevity in commercial beef cow–calf operations remains elusive. The telomere dynamics of beef cattle may vary from what has been reported for commercial dairy operations. It is common practice in the modern dairy industry for the calf to be removed shortly after birth to allow for maximum milk yield from the cow. This eliminates the very well-established stress of caregiving that is known to result in shortened telomere lengths in humans, avian species, and mice [24,25,26,27,28]. The results from the present study suggest that the stress of parturition and successfully raising the first calf of a cow’s life may be responsible for the attenuation of the TL in leukocytes (white blood cells, WBC) in cows.

## 2. Materials and Methods

All procedures followed the Guide for the Care and Use of Agricultural Animals in Research and Teaching [29] and were approved by the Texas A&M AgriLife Research Agricultural Animal Care and Use Committee (Animal Use Protocol 2017-035A).

### 2.1. Experimental Design

Multiparous cows (n = 11) and primiparous cows (n = 8; heifers) that were 4 years of age and were determined to be pregnant via artificial insemination were used in this study. These females were from the registered purebred Brahman *(Bos indicus)* research herd located at the Texas A&M AgriLife Research and Extension Center in Overton, TX, USA (approximately 32°16′15.00″ N; 94°58′20.99″ W). The cows were calm to intermediate in temperament. The cattle were maintained on pastures consisting of Coastal bermudagrass (*Cynodon dactylon*) overseeded with Maton rye (*Secale cereal* L.) and Nelson ryegrass (*Lolium multiflorum* Lam.) from 1 March 2018 to 26 May 2018. All multiparous cows were undergoing the second gestation of their lifetime, whereas primiparous cows were undergoing their first gestation. In addition to pasture and free choice Coastal bermudagrass hay, cows and heifers were fed approximately 3.6 kg of a 3:1 corn/corn gluten grain mix per head daily for the duration of the experiment. The cattle were maintained in a calving pasture that is adjacent to the field laboratory. Attached to the field laboratory is the covered cattle handling race set-up that was used to confine or restrain the cattle to obtain body weight and blood samples. Early in the morning of the collection days, the cattle scheduled for sampling were moved quietly at a walking pace for approximately 500 m to enter the race system. The 4-year-old cattle in this research resource herd were accustomed to the herdsman and the handling associated with the routine animal health management procedures that occur in this facility. The cows progressed through the race without issue. The width of the squeeze chute and the headgate were adjusted to accommodate the size of the cows and to facilitate animal and human safety during the collection of blood samples via jugular venipuncture.

Blood samples were collected from the cows and heifers via jugular venipuncture. Cows and heifers were weighed, and body condition score (BCS) was recorded 28 days prior to calving (Day − 28) and at 7 days and 28 days post-calving (Day + 7 and Day + 28 post-calving, respectively; Table 1). Evaluation of BCS on a scale of 1 to 9 (1 = thin, 9 = fat) [30,31] was performed and recorded by a trained professional with over 40 years of experience.

At the time of parturition, cows and heifers were monitored for duration of labor, calving ease, and retained placenta. Cows and heifers were monitored at the onset of observable signs of parturition such as relaxation of the pelvic ligaments, swollen vulva, and fluid discharge. The timing of the duration of labor began at signs of hard labor, such as the cow laying down with observable contractions, to the expulsion of the calf. Calf birth weight and sex were recorded within 24 h of parturition.

### 2.2. White Blood Cell (WBC) Count with Differential

Based on individual calving dates, blood samples were collected from the cows and heifers via jugular venipuncture (20 mL total per animal) Day − 28 prior to calving as well as on Day + 7 and Day + 28 after calving utilizing a sterile 18-gauge needle and sterile 10-mL vacuum tubes containing EDTA (Becton Dickinson & Co., Franklin Lakes, NJ, USA). A Neubauer hemacytometer (Thermo Fisher Scientific, Houston, TX, USA) was used to estimate the total white blood cell (WBC) number in the blood samples collected pre- and post-partum from both groups of cows. A WBC count with differential was performed with Giemsa staining (Thermo Fisher Scientific, Houston, TX, USA) of whole blood smears of each blood sample on separate glass microscope slides to monitor possible pre- and post-partum alterations in the proportion of the types of WBCs [32,33,34]. The identity of each slide was coded to blind the technician who used a binocular microscope to review the Giemsa-stained slides [35]. There was a two-fold purpose to focus on these parameters of the nucleated WBC in the assessment of TL in the Parity 1 and Parity 2 cows of this study. First, whether time and parity affected the TL would be difficult to discern if a cow had experienced inflammation/infection (i.e., an increased WBC number) during the study. Second, awareness of alterations in the proportion of the type of WBC present in the blood sample would be informative as to the cellular source of telomeric DNA.

### 2.3. White Blood Cell (WBC) Isolation and DNA Extraction

Whole blood was centrifuged at 2675× *g* for 30 min at 5 °C. After centrifugation, the WBC layer located between the red blood cell (RBC) and serum interface was aspirated using a pipette and transferred into a 1.5 mL storage tube. The WBCs were then cleaned of any remaining RBC and serum utilizing an RBC lysis buffer containing EDTA and water. The WBCs were mixed with the RBC lysis buffer for 5 min and micro-centrifuged for 5 min at 5000× *g* at room temperature. The remaining RBC, serum, and RBC lysis buffer were aspirated from the top of the WBC pellet. The WBC pellet was then stored in sterile, nuclease-free microfuge tubes at −80 °C.

Extraction of DNA was performed utilizing spin column GeneJET Genomic DNA Purification Kits (Thermo Scientific; Waltham, MA, USA). Prior to extraction, frozen WBCs were crushed via liquid nitrogen utilizing a mortar and pestle. Twenty milligrams of the crushed WBC pellet was used for extraction. The remainder of the crushed WBC pellet was returned to the sterile microfuge tube for storage at −80 °C. Quality control of extracted DNA was conducted at the Genomics Core Lab of the Texas A&M University Institute for Genome Sciences and Society. Quality control criteria were as outlined in the study by Seeker et al. [36] as follows: yield > 20 ng/µL; gel integrity score < 3; 260/280 absorbance ratio > 1.7; and 260/230 absorbance ratio > 1.8. The DNA samples that passed quality control were aliquoted and diluted in low-binding tubes to 10 ng/µL utilizing nuclease-free water, and stored at −80 °C until used in PCR procedures.

### 2.4. Quantitative PCR

Quantity of telomere sequences was determined utilizing real-time quantitative PCR methods utilizing the ratio of telomere to bovine beta-2-globulin (β2G) genes. Custom primers for amplifying bovine telomere sequences and bovine β2G (Table 2; defined by Cawthon et al. [37] and Brown et al. [12]) were designed and purchased from Integrative DNA Technologies (IDT; Coralville, IA, USA). The bovine β2G gene was selected as a relatively constant housekeeping gene that operates as a control gene for determining relative quantity of telomere [12]. The master mix for the telomere and β2G reaction contained 10 µL 2X PowerUp SYBR Green Master Mix (Applied Biosystems; Foster City, CA, USA), 1 µL of bovine telomere primers (forward and reverse, 500 nmol final concentration each), 0.6 µL of β2G primers (forward and reverse, 300 nmol final concentration each), and 4.8 µL nuclease free water. Master mix was loaded prior to 20 ng/µL of DNA. Standard double-stranded DNA oligonucleotides of bovine telomere and β2G were designed and purchased from IDT (Table 3). Six-step 1:10 serial dilutions of both bovine telomere standard (10^12^–10^7^) and bovine β2G (10^9^–10^4^) to create standard curves were immediately generated prior to loading. Samples were randomly allocated on a 384-well qPCR plate and amplified in triplicate 20 µL reactions. A negative control containing water in place of DNA was included to monitor for contamination of master mix. A real-time system thermocycler (BioRad; Hercules, CA, USA) was programed with multiplex settings defined by Brown et al. [12]. Cycle threshold (C_q_) value triplicates were checked for consistency.

Triplicates were included if the cycle difference was less than 0.25 cycles. Single replicates with differences larger than 0.25 cycles were removed if an obvious outlier could be identified, and the remaining wells were included. Samples with remaining wells with differences larger than 0.25 cycles were excluded from analysis. Starting quantities (SQ), based on the six-step dilution series of either β2G or telomere standards, were averaged for samples with adequate C_q_ replicate differences. Copy number of telomere sequence (5′-TTAGGG-3′) and β2G gene is represented by the corresponding SQ. A ratio of telomere SQ and β2G SQ was utilized to determine T/S ratio, which can be interpreted as TL [37].

### 2.5. Statistical Analysis

Statistical analyses were conducted using mixed linear models with the MIXED procedure of SAS 9.4 (SAS Inst., Inc., Cary, NC, USA). All T/S ratio measurements were analyzed after log_10_ transformation to normalize their distribution. Parity (first or second) was the fixed effect of interest. Other investigated fixed effects included calf birth weight, duration of labor, and calving ease and their interactions, and the BW, BCS, and CBC were evaluated as linear covariates. Random effects included sire and day repeated with cow as the subject.

## 3. Results

No cows were observed to exhibit health issues during the period of this study. There was no indication of unobserved inflammation or infection, as the estimated total WBC did not differ (*p* > 0.10) from Day − 28 to Day + 28 within Parity 1 and Parity 2 cows, nor did the total WBC differ between the two parity groups (*p* > 0.10). Specifically, the total WBC on Day − 28, Day + 7, and Day + 28 were 6.9 ± 2, 7.6 ± 0.9, and 6.9 ± 1.7 × 10^3^/μL, respectively, for the Parity 1 cows. Similarly, for the Parity 2 cows, the total WBC on Day − 28, Day + 7, and Day + 28 averaged 6.7 ± 1.8, 8.9 ± 1.9, and 6.8 ± 1.5 × 10^3^/μL, respectively. These total WBC values are within the normal reference interval of 4-to-12 × 10^3^/μL for healthy beef cattle [38]. The number of lymphocytes and neutrophils were not statistically different from Day − 28 to Day + 28 and did not differ between the parity groups. During the period of this study, a similar range in the lymphocytes was noted for the Parity 1 cows (3.9 ± 1 to 4.9 ± 0.7 × 10^3^/μL) and Parity 2 cows (3.6 ± 0.8 to 4.6 ± 0.9 × 10^3^/μL). The number of neutrophils was consistent over time for the Parity 1 cows (2.0 ± 0.7 to 2.3 ± 0.9 × 10^3^/μL) and Parity 2 cows (2.3 ± 0.5 to 2.6 ± 0.7 × 10^3^/μL). The values observed for the lymphocytes, neutrophils, monocytes, eosinophils, and basophils were also within the normal reference interval [38]. Based on these data, there was not an inflammatory event, and the TL measurements reported for the cows in this study are independent of the changes in the number of WBC types.

**Figure 1:**
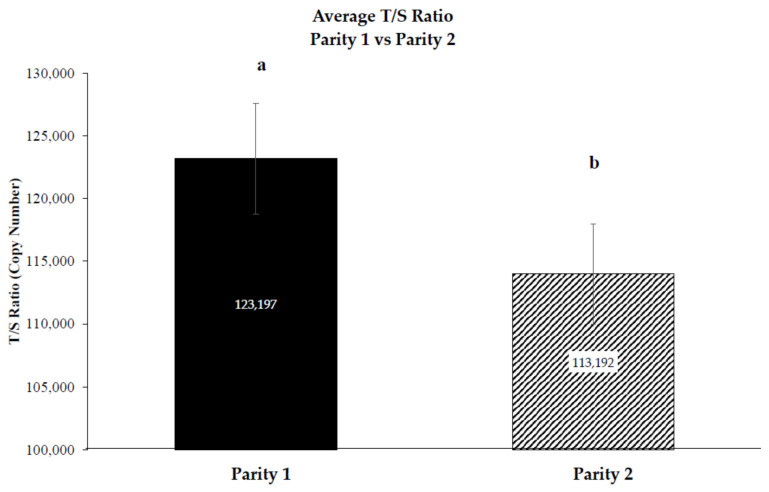
The effect of parity (1, 2) on average Brahman cow telomere length as represented by the T/S ratio [36]. Age-matched Brahman cows who have had their second calf of their lifetime, compared to cows who had their first calf, have a significant reduction in telomere length (TL) (*p* = 0.02) of 9225 sequences or approximately a 7.2% reduction. Error bars represent SE. a,b Significant difference (*p* < 0.02).

Nineteen cows were included in the analysis. Two calves died between the Day + 7 and Day + 28 post-calving sampling days due to inclement weather. Therefore, the d + 28 post-calving blood samples from a Parity 1 dam and a Parity 2 dam were excluded from analysis. Two samples that did not meet the PCR C_q_ replicate criteria were excluded from analysis. A reduction in the TL was detected (*p* = 0.02) between Parity 1 (123,197 ± 4426) and Parity 2 (113,972.5 ± 3973), with the Parity 2 cows having approximately 9225 less copies of telomere sequences (Figure 1).

**Figure 2:**
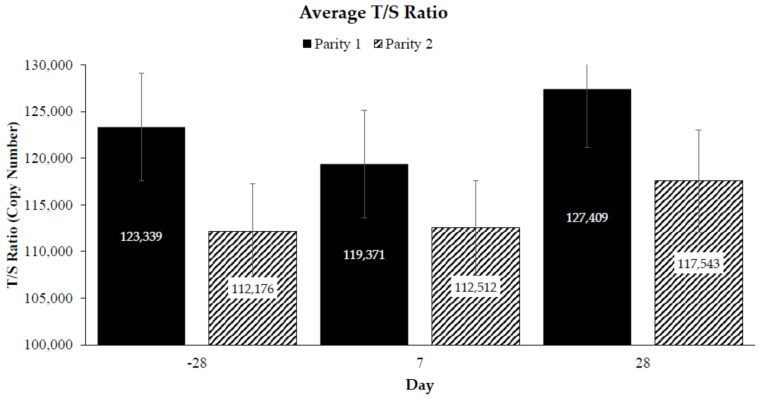
The effect of sample day (Day − 28pre-calving, Day + 7, and Day + 28 post-calving) and parity (1, 2) on average Brahman cow telomere length as represented by the T/S ratio [38]. The interaction of sample day and parity did not impact the telomere length (*p* = 0.76). Error bars represent SE.

No statistical significance was detected by day (*p* = 0.23) and in the interaction of parity and day (*p* = 0.76) (Figure 2).

The average duration of labor for the Parity 1 females was 188 min (range: 50 to 450 min). The average duration of labor for Parity 2 was 138 min (range: 90 to 340 min). A trend was observed between the duration of labor and the TL (*p* = 0.06). The Pearson correlations show a positive correlation (coefficient 0.46) between the duration of labor and the T/S ratio (prob > |r| 0.0008). The regression of the TL on the BW, BCS, and CBC were not significant (*p* = 0.57; *p* = 0.38; *p* > 0.1, respectively). Additionally, a statistical significance in the TL was not observed for the calving ease (*p* = 0.19) or the calf birth weight (*p* = 0.79) as linear covariates.

## 4. Discussion

The primiparous and multiparous cows differed in the TL overall despite the interaction of parity and day not being significant in this study. It was not surprising that parity had a significant negative effect on the TL. Parental effort and parity influence the TL in a variety of species. Specifically, parity, lactation, and parental effort have a negative effect on the TL in species that are as varied as humans [24,25,39], birds [27,28,40], and cattle [12,13,41]. The effect of parity between nulliparous and multiparous species on the TL was reported in women. Specifically, multiparous women had 4.2% less telomeric sequences than nulliparous women [39]. In the present study, multiparous cows had approximately a 7.5% reduction in the T/S ratio compared to the primiparous cows. This is an interesting finding, considering that a productive cow is typically expected to have 7.5 calves in her lifetime. If this trend is continued through a cow’s productive lifespan of 7.5 calves, then there would be approximately a 52.5–60% reduction in the total TL. This suggests that a cow may begin to decline in productivity when the TL is reduced by 52.5–60% from her first calving. This finding is particularly interesting, considering that beef heifers (5/8 Red Angus, 3/8 Simmental) born to older dams (≥7 years of age) were found to have reduced productivity and retention in the herd compared to heifers born to moderate age (4 to 6 years of age) or young age (2 to 3 years of age) dams [42]. It is possible that the telomere dynamics of age and parity may influence the differences in progeny longevity between the dam age groups [43]. The evaluation of the calf telomere length compared to the dam age and parity could be an interesting next step in optimizing the selection of replacement heifers with increased longevity and productivity. Recently, Froy et al. [44] determined that a variation in the TL has a genetic basis (heritability estimate of approximately 0.20) in wild Soay sheep. Further, their finding suggests that an animal’s average TL at a young age may provide insight regarding longevity (and, by extension, productivity) due to the individual’s stress responsiveness or resilience to stressors encountered with age.

The lack of differences in the interaction of day and parity was unexpected. Parturition is a complex cascade of glucocorticoid signaling and changes in hormone profiles that are suggested in the literature to impact the activity of telomerase and to directly damage telomeres [45,46,47,48]. However, the lack of differences in the TL pre-partum and post-partum may be attributed to the protective effects of estrogens. The post-partum resumption of the ovarian function and the return to estrus means that the plasma concentrations of estrogens will return to normal levels of the estrous cycle [49]. Estradiol is known to bind to the promoter region of the telomerase reverse transcriptase (TERT) gene and upregulate telomerase activity [45]. It is possible that this is a mechanism that is in place to protect telomeres after the physiological stress of gestation and parturition.

The overall difference in the TL between Parity 1 and Parity 2 may be related to lactation and the psychological stress of caregiving to the calf. Laubenthal et al. [41] found a strong positive correlation between the initial leukocyte quantity of telomeres (qT) in early lactation and the extent of qT attrition in late lactation in Holstein cattle. However, this was limited to cows with initially high qT values, but not in those with initially low qT values. This indicates that the protective mechanisms of telomeres act preferentially on lower qT values or “shorter” telomeres in dairy cattle. However, Seeker et al. [13] did not detect a difference in the relative length of telomeres (RLTL) between two genetic lines for high milk yield and control cows but found that milk yield significantly affected the lifespan. Although Brown et al. [12] did not evaluate the milk yield, they reported a negative linear relationship between age and qT, as well as a relationship between qT and culling status in cows. This conflicted with Seeker et al. [13], who did not report an age-related decline in TL in cows up to 6 years of age but acknowledged that geriatric (13–14 years of age) cows were included in the study by Brown et al. [12]. These conflicting studies suggest that parity and lactation in dairy cattle may or may not influence telomere dynamics. It is important to note that this previous research was conducted on dairy cattle, and in the dairy industry, cows are required to produce a calf but must off-set their maintenance costs with their milk yield. In the previously cited studies, the cows were not responsible for raising their calf. However, in the beef cow–calf industry, cows are responsible for raising their calves to weaning. This suggests that the reduction in the TL in the present study was most likely driven by the physiological stress of caregiving to the calf and ensuring calf survival. This is consistent with detection of a strong relationship between varying caregiving stress scenarios and TL in humans [24,25].

The positive trend observed in the duration of labor in relation to the TL was unexpected. Parturition is a physiologically strenuous event, and parity was found to negatively impact the TL, as stated above. Perhaps endocrine signaling related to the onset of labor may play a role in telomere dynamics. During parturition in ruminants, the onset of labor is dependent upon the activation of the fetal hypothalamic–pituitary–adrenal axis [50]. This increases the plasma concentration of cortisol to induce the activity of the enzymes17α-hydroxylase, 17–20 desmolase, and aromatase in the placenta, which results in the biosynthesis of estradiol relative to progesterone [50]. While cortisol may inhibit telomerase, it is possible that the biosynthesis of estrogens during labor may impact telomerase activity as well [9,45]. It is important to note that the duration of labor was measured by observation, and signs of labor are subjective to the observer. The true duration of time that the cows spent in labor may vary from what was recorded, which may limit this analysis.

Since the inception of our study with cattle, pilot studies by Panelli et al. [51,52] compared the leukocyte TL of women whose babies were delivered by vaginal or cesarian birth. Although the leukocyte TL did not vary pre-partum in the early and late trimesters of gestation, there was a trend of a shorter TL in women who reported an elevated degree of stress. However, within 1 day of delivery, relative to the vaginal delivery group, the leukocyte TL was shorter for the women in the cesarian delivery group (data pooled from the patients whose labor complications required surgery and the patients who elected surgery). The potential influence of gestational and parturition complications on the telomere dynamics of dams and their progeny constitutes a new research avenue to elucidate the longitudinal effects of prenatal and early life stimuli [43,53,54,55].

## 5. Conclusions

In summary, the parity of Brahman cows was a significant predictor of their telomere attrition when controlled for age. While no changes in the TL were detected between Day − 28 and Day + 28 for Parity 1 and Parity 2, the difference between the parities alone was most likely attributed to the parental effort of caring for a calf. The telomere length shows a tendency to extend with an increase in the time spent in hard labor in this study. Changes in hormone signaling during parturition, particularly estrogen, may act as protective mechanisms in a time of increased physiological stress. However, this may be a unique circumstance of studying primiparous Brahman cows that are undergoing the stress of birth and caregiving for the first time. Studying age-matched cows of varying parities beyond two calves and the role of telomerase is necessary to fully understand the relationship between parturition, cow longevity, and telomere attenuation. Future investigation of the linkage of telomere maintenance with gene expression, health, productivity, and longevity of beef cattle should encompass appropriate tissue-specific analyses, particularly to increase the comprehension of the potential effects of environmental stressors on the intrauterine or epigenetic programming of the three embryonic germ layers of calves [56,57,58,59,60].

## Figures and Tables

**Table 1:** Body weight (BW) and body condition score (BCS) means (SEM) for Parity 1 and Parity 2 by sample day.

Sample Day				
	Parity	Day − 28	Day + 7	Day + 28
BW, kg	Parity 1	499 ± 15	507 ± 17	513 ± 9
	Parity 2	526 ± 14	521 ± 15	547 ± 10
BCS	Parity 1	7.00 ± 0.17	6.75 ± 0.21	6.63 ± 0.16
	Parity 2	6.90 ± 0.22	6.73 ± 0.22	6.68 ± 0.23

**Table 2:** Nucleotide sequence of bovine-specific primers used in quantitative real-time reverse transcription PCR to determine expression of target genes.

Target Gene	Gene Name	IDT Ref# ^1^	Primer	Sequence
Telomere	Telomere	207839206 207839207	Forward Reverse	5′-ACACTAAGGTTTGGGTTTGGGTTTGGGTTTGGGTTAGTGT-3′ 5′-TGTTAGGTATCCCTATCCCTATCCCTATCCCTATCCCTAACA-3′
β2G	Beta-2-Globulin	207839208 207839209	Forward Reverse	5′-CGGCGGCGGGCGGCGCGGGCTGGGCGGGAAGGCCCATGGCAAGAAGG-3′ 5′-GCCGGCCCGCCGCGCCCGTCCCGCCGCTCACTCAGCCAC-AAAGG-3′

^1^ Integrative DNA Technologies (IDT; Coralville, IA, USA).

**Table 3:** Nucleotide sequences of bovine-specific oligonucleotides utilized to create standard curves for expression of target genes in quantitative real-time PCR.

Standard Name	IDT Ref # ^1^	Strand	Sequence
Bovine Telomere	206903826	Sense	5′-TTAGGGTTAGGGTTAGGGTTAGGGTTAGGGTTA GGGTTAGGGTTAGGGTTAGGGTTAGGGTTAGGGTTAGGGTTAGGGTTAGGG-3′
		Antisense	5′-CCCTAACCCTAACCCTAACCCTAACCCTAACC CTAACCCTAACCCTAACCCTAACCCTAACCCTAACCCTAACCCTAA-3′
Bovine Beta-2-Globulin	206903823	Sense	5′-GGTGAAGGCCCATGGCAGAAGGTGCTAGATT CCTTTAGTAATGGCATGAAGCATCTCGATGACCTCAAGGGCACCTTTGCGCTGAGTGAGCTG-3′

^1^ Integrative DNA Technologies (IDT; Coralville, IA, USA).

## Data Availability

The data will be made available upon reasonable request.
